# The Identification and Characterization of Novel N-glycan-based Biomarkers in Gastric Cancer

**DOI:** 10.1371/journal.pone.0077821

**Published:** 2013-10-17

**Authors:** Long Liu, Bing Yan, Junlong Huang, Qunhao Gu, Lina Wang, Meng Fang, Jianpeng Jiao, Xiaoqiang Yue

**Affiliations:** 1 Department of Traditional Chinese Medicine, Changhai Hospital, Second Military Medical University, Shanghai, China; 2 Department of Traditional Chinese Medicine, Changzheng Hospital, Second Military Medical University, Shanghai, China; 3 Department of Nautical Medicine, Laboratory of Stress Medicine, Faculty of Naval Medicine, Second Military Medical University, Shanghai, China; 4 Department of General Surgery, Yueyang Hospital, Shanghai University of Traditional Chinese Medicine, Shanghai, China; 5 Department of Laboratory Medicine, Eastern Hepatobiliary Surgery Hospital, Second Military Medical University, Shanghai, China; The University of Kansas Medical center, United States of America

## Abstract

**Background and Aims:**

To identify and validate N-glycan biomarkers in gastric cancer (GC) and to elucidate their underlying molecular mechanism of action.

**Methods:**

In total, 347 individuals, including patients with GC (gastric cancer) or atrophic gastritis and healthy controls, were randomly divided into a training group (n=287) and a retrospective validation group (n=60). Serum N-glycan profiling was achieved with DNA sequencer-assisted/fluorophore-assisted carbohydrate electrophoresis (DSA-FACE). Two diagnostic models were constructed based on the N-glycan profiles using logistic stepwise regression. The diagnostic performance of each model was assessed in retrospective, prospective (n=60), and follow-up (n=40) cohorts. Lectin blotting was performed to determine total core-fucosylation, and the expression of genes involved in core-fucosylation in GC was analyzed by reverse transcriptase-polymerase chain reaction.

**Results:**

We identified at least 9 N-glycan structures (peaks) and the levels of core fucose residues and fucosyltransferase were significantly decreased in GC. Two diagnostic models, designated GCglycoA and GCglycoB, were constructed to differentiate GC from control and atrophic gastritis. The areas under the receiver operating characteristic (ROC) curves (AUC) for both GCglycoA and GCglycoB were higher than those for CEA, CA19-9, CA125 and CA72-4. Compared with CEA, CA19-9, CA125 and CA72-4, the sensitivity of GCglycoA increased 29.66%, 37.28%, 56.78% and 61.86%, respectively, and the accuracy increased 10.62%, 16.82%, 25.67% and 28.76%, respectively. For GCglycoB, the sensitivity increased 27.97%, 35.59%, 55.09% and 60.17% and the accuracy increased 21.26%, 24.64%, 31.40% and 34.30% compared with CEA, CA19-9, CA125 and CA72-4, respectively. After curative surgery, the core fucosylated peak (peak 3) and the total core fucosylated N-glycans (sumfuc) were reversed.

**Conclusions:**

The results indicated that the diagnostic models based on N-glycan markers are valuable and noninvasive alternatives for identifying GC. We concluded that decreased core-fucosylation in both tissue and serum from GC patients may result from the decreased expression of fucosyltransferase.

## Introduction

Gastric cancer (GC) is the fourth most prevalent cancer and the third leading cause of cancer-related death, with an incidence of approximately 930,000; annually, it is responsible for over 700,000 deaths worldwide, and the five-year survival rate is 20-30% [1]. For patients with GC, survival is dictated by the pathologic stage of the disease at the time of diagnosis. Unfortunately, the common symptoms of GC are not specific to the disease, and early stage GC may not cause noticeable symptoms. Despite increasing knowledge of the molecular mechanisms that regulate malignant transformation and metastasis, the overall survival rate of patients with GC has not improved significantly. Several molecules have been recommended as GC biomarkers, including carcinoembryonic antigen (CEA) for postoperative surveillance, carbohydrate antigen 19-9 (CA19-9), carbohydrate antigen 125 (CA125) and carbohydrate antigen 72-4 (CA72-4) [2-6]. However, none of these tumor markers has demonstrated sufficient sensitivity or specificity for diagnosing GC at an early stage. Alternatively, gastroscopy increases the rate of definitive diagnoses, but the diagnostic value of gastroscopy is limited by cost, risks, and inconvenience. Therefore, there is an urgent need for the development of noninvasive biomarkers that enable the early detection of GC.

Increasing evidence suggests the alteration of N-linked glycan could be regarded as a potential biomarkers for diagnosing cancer. Previous studies observed a significant changes of N-linked glycan in different cancers and cancer cell lines which includes pancreatic cancer, breast cancer, prostate cancer, ovarian cancer and liver cancer [7-11]. Additional analysis in GC also uncovered that free complex-type N-glycans accumulated in MKN7 and MKN45 cell lines [12]. However, the fluctuation and variation of N-linked glycan in GC patients are still largely unknown.

In our previous studies using DNA sequencer-assisted/fluorophore-assisted capillary electrophoresis (DSA-FACE), we demonstrated that a branching α-1,3-fucosylated triantennary glycan and a biantennary glycan were highly specific and sensitive candidate HCC biomarkers [13]. In the current study, we utilized DSA-FACE to retrospectively profile serum N-glycans in samples from patients with GC or atrophic gastritis and from healthy individuals. We characterized the identified GC N-glycan markers with receiver operating characteristic (ROC) curves and validated the markers in prospective and follow-up cohorts. Our objective was to identify a promising biomarker for predicting and detecting GC with improved specificity and sensitivity.

## Materials and Methods

### 1.1: Retrospective cohort

The study protocol was approved by The Chinese Ethics Committee of Human Resources at the Second Military Medical University. Written informed consent was obtained from the patients and the healthy controls.

In total, 247 patients with GC (n=138) or atrophic gastritis (n=109) were recruited between 2010 and 2012. The diagnoses for all the enrolled patients were histopathologically confirmed by 2 pathologists at Changhai Hospital, Second Military Medical University (Shanghai, China). In the control group, 128 age- and sex-matched healthy volunteers (disease-free) were enrolled during the same time period. The mean age and the sex distribution were matched in the 3 groups. A summary of the patient data is provided in Table 1. GC patients who received preoperative radiotherapy, chemotherapy, or chemoradiotherapy were excluded from the study.

**Table 1:** Characteristics of the training group.

	Mean ± SD or No. (%)		
Characteristic	Control (n=128)	Atrophic Gastritis (n=109)	GC (n=138)
Age, y	50.10±5.95	52.11±6.29	51.01±6.01
Men	75 (58.59)	60 (55.05)	70 (50.72)
CEA-positive	5 (3.91)	23 (21.10)	62 (44.93)
CA19-9-positive	11 (8.59)	20 (18.35)	53 (38.41)
CA125-positive	7 (5.47)	10 (9.17)	28 (20.29)
CA72-4-positive	8 (6.25)	12 (11.01)	22 (15.94)
TNM stage			
I			22 (15.94)
II			28 (20.29)
III			63 (45.65)
IV			25 (18.12)

Abbreviations: GC, gastric carcinoma; SD, standard deviation.

A total of 60 patients (20 in each group) were randomly selected from the 3 groups described above for retrospective verification; the remaining patients (n=315) were included in the training set to construct the diagnostic model. During the 2 years of the study, 40 of the 138 patients with GC in the training group were monitored before and after curative surgery, based on availability. The training group included 118 patients with GC, 89 patients with atrophic gastritis, and 108 healthy controls.

Laboratory and clinical data for all the participants were obtained from clinical medical records, pathology reports, and personal interviews. The data collected included sex, age, and gastric cancer features (such as tumor location, histologic grade, depth of invasion, and lymph node metastasis). Serum samples were obtained before surgical resections; these samples were collected from whole blood using a standard protocol, centrifuged at 10,000xg for 20 minutes, and stored at -80°C. Disease progression in the GC patients was classified according to the seventh edition of The American Joint Committee [14]: 20 patients (16.95%) had stage I disease, 25 (21.17%) had stage II disease, 54 (45.76%) had stage III disease, and 19 (16.10%) had stage IV disease.

### 1.2: Prospective cohort

To evaluate the predictive value of the models established in the retrospective study described above, we prospectively investigated an additional cohort (n=60) of patients with GC (n=20) or atrophic gastritis (n=20) and of healthy individuals (n=20) from May 2012 to November 2012 at the same hospital. The procedures and strategies were the same as those described above.

### 1.3: Tissue samples

Tissue samples were obtained from 20 of the 138 GC patients; 2 slices, a tumor and an adjacent tissue sample, were taken. The tissue samples (approximately 1 cm^3^) were immediately frozen at -80°C and subjected to reverse transcriptase-polymerase chain reaction (RT-PCR) and lectin blotting. All the tissues were used in accordance with the Institutional Review Board Regulations of the Second Military Medical University.

### 1.4: Routine detection of tumor markers

Routine tumor marker assays were performed using standard methods and reagents. CEA and CA19-9 levels were determined on an Abbott I2000, and CA72-4 and CA125 levels were measured using a Roche Cobas E601. The cut-off levels recommended by the manufacturer for CEA, CA19-9, CA125 and CA72-4 were 5.0 μg/L, 39 U/L, 40 U/ml and 9.8 U/ml, respectively. The assays were performed in the Department of Laboratory Medicine, Changhai Hospital, the Second Military Medical University, Shanghai.

### 1.5: Serum protein N-Glycan profiling

Serum protein N-glycan analyses were performed as previously described [13]. Briefly, the N-glycans in 2 μL of serum were released from the proteins with peptide-N-glycosidase F (PNGase F) (New England Biolabs, Boston, MA) labeled with 8-aminonaphtalene-1,3,6-trisulphonic acid (Invitrogen, Carlsbad, CA). Sialic acid was removed using *arthrobacter ureafaciens* sialidase (Roche Bioscience, Palo Alto, CA), and the processed samples were analyzed using DSA-FACE technology on a capillary electrophoresis-based ABI3130 Genetic Analyzer (Applied Biosystems, Foster City, CA). The 9 highest peaks that were detected in all the samples (accounting for >90% of the total serum N-glycans) were analyzed using GeneMapper (Applied Biosystems). Each N-glycan structure was described numerically by normalizing its height to the sum of the heights of all the peaks, and we analyzed these data using SPSS 18.0 statistical software (SPSS Inc., Chicago, IL).

### 1.6: Tissue protein extraction and lectin blotting

The tissues were homogenized using a mortar and pestle and resuspended in lysis buffer containing a protease inhibitor cocktail (Roche Diagnostics, Meylan, France). The unlysed fraction was removed by centrifugation (twice at 12000xg for 10 minutes at 4°C). The concentration of soluble protein was determined using the BioRad assay (BioRad, Marnes-la-Coquette, France), and the samples were stored at -80°C.

In total, 25 μg of serum protein or 50 μg of protein extracted from frozen tissue was separated by 10% sodium dodecyl sulfate-polyacrylamide gel electrophoresis. The gels were stained with CBB G250, or the proteins in the gel were transferred to a nitrocellulose membrane (Whatman/Schleicher & Schuell, Versailles, France) to detect core-fucosylated proteins. The membranes were blocked overnight at 4°C with 5% bovine serum albumin in Tris-buffered saline (TBS: 140 mM NaCl and 10 mM Tris-HCl) and then incubated for 1 hour at room temperature with 5 μg/mL of biotinylated lens culinaris agglutinin A (LCA) (Vector Laboratories, Burlingame, CA) in TBS containing 0.05% Tween-20 (TBST). After 4 washes of 10 minutes each with TBST, the membranes were incubated with IRDye 800CW Streptavidin (1:10,000; LI-COR Biosciences, Lincoln, NE) for 1 hour at room temperature, washed 4 times with TBST, and developed using the Odyssey Infrared Imaging System (LI-COR Biosciences). Purified albumin (Sigma, St. Louis, MO) was used as a negative control for the lectin blot.

### 1.7: Total RNA extraction from tissue and quantitative real-time PCR

RNA was extracted from frozen tissues using a Qiagen RNeasy mini kit according to the manufacturer’s instruction (QIAGEN GmbH, Hilden, Germany). The purity and concentration of the RNA were determined using a spectrophotometer (Eppendorf, Hamburg, Germany). cDNA was synthesized from 2 μg of total RNA using a reverse transcription reagent (Toyobo, Osaka, Japan). The primers were designed using Primer Express (Applied Biosystems), and the sequences are presented in Table 2.

**Table 2:** Polymerase chain reaction primer pair sequences.

Gene	Forward Primer (5’-3’)	Reverse Primer (5’-3’)
Fut8	5'CCTGGCGTTGGATTATGCTCA 3'	5'CCCTGATCAATAGGGCCTTCT 3'
GDP-Tr	5'CTGCCTCAAGTACGTCGGTG 3'	5'CCGATGATGATACCGCAGGTG 3'
GAPDH	5'ATGGGGAAGGTGAAGGTCG 3'	5'GGGGTCATTGATGGCAACAATA 3'

Abbreviations: A, adenosine; C, cytidine; G, guanosine; T, thymidine; Fut8, fucosyltransferase; GDP-Tr, guanosine diphosphate-fucose transporter

The cDNAs were amplified in an Applied Biosystems 7300 Real-Time PCR machine in a total reaction volume of 20 μL that contained 10 μL of 2X Fast SYBR Green Master Mix (Applied Biosystems, includes Fast-Start Taq DNA polymerase reaction buffer), the deoxynucleotide triphosphate mix (including deoxyuridine triphosphate, SYBR Green I dye, and MgCl2), and 2 μL of the primers for each gene (at a final concentration of 0.5 μΜ each). Each reaction was performed in triplicate. The PCR cycling conditions were as follows: denaturation at 95°C for 5 minutes followed by 40 cycles of 95°C for 15 seconds, 59°C or 55°C for 15 seconds, and 72°C for 45 seconds.

The relative expression of α-1,6-fucosyltransferase (Fut8) and guanosine diphosphate (GDP)-fucose transporter (GDP-fuc-Tr) in each sample was normalized to the expression of the GAPDH housekeeping gene by subtracting the threshold cycle (Ct) value of GAPDH from that of Fut8 or GDP-Tr (∆Ct). The fold difference was calculated by subtracting the ∆Ct of the test sample from that of the control sample to obtain the ∆∆Ct and subsequently the 2^-∆∆Ct. Threshold cycle values beyond 40 cycles were considered below the detectable level. Melt curves were obtained for each reaction to guarantee that a single product was amplified.

### 1.8: Statistical analysis

All the quantitative variables were expressed as the mean ± standard deviation, unless otherwise indicated. The quantitative variables were compared using Student’s t-test, analyses of variance, or nonparametric tests. Pearson’s correlation coefficients and the associated probabilities (P) were used to evaluate the correlations between the parameters; Spearman’s correlation coefficients were calculated for ordinal categorical variables. Novel glycan biomarkers were identified and characterized based on a forward stepwise logistic regression analysis. The diagnostic performance of individual biomarkers and of the diagnostic models was evaluated using ROC curve analysis. The sensitivity, specificity, positive predictive value (PPV), negative predictive value (NPV), and accuracy were calculated using optimal cutoff values selected from the ROC curves. All the reported P values are 2-tailed, and P values <0.05 were considered statistically significant. The statistical analyses were performed using SPSS 18.0 for Windows (SPSS Inc.).

## Results

### 2.1: Different N-Glycan profiles in patients with GC or atrophic gastritis and in healthy controls

**Figure 1:**
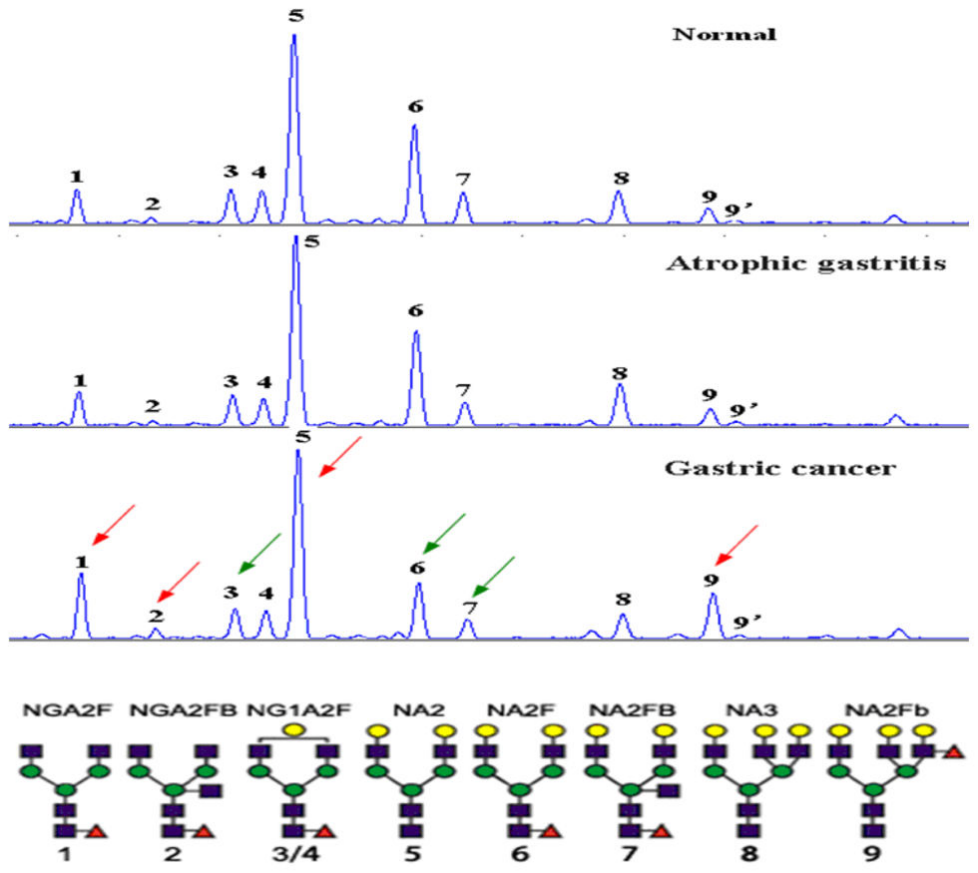
A typical desialylated N-glycan profile of serum protein. At least 9 peaks can be identified. Peaks 1, 2, 5 and 9 increased (red arrows), and peaks 3, 6 and 7 decreased (green arrows) in gastric carcinoma compared with normal controls. The structures of the N-glycan peaks are shown below the chart. The open circles indicate b-linked galactose; the triangles, a/b-1,3/6-linked fucose; and the solid circles, a/b-linked mannose.

Using DSA-FACE technology, we examined the N-glycan profiles in patients with GC (n=118) or atrophic gastritis (n=89) and in healthy individuals (n=108). We quantified and statistically compared the peaks in the 3 groups. At least 9 N-glycan structures (peaks) were identified in all the samples (Figure 1). 

**Figure 2:**
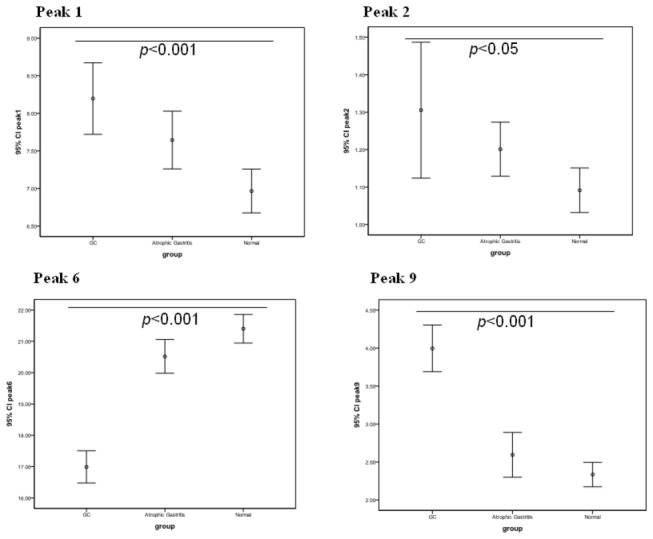
The changes in N-glycan levels for the 3 groups. In the gastric cancer (GC) group, the levels (represented as 95% confidence intervals [CIs]) of agalacto, core-α-1,6-fucosylated biantennary glycan (NGA2F,peak1), core-α-1,6-fucosylated bisecting biantennary glycans (NGA2FB, peak2) and branching α-1,3-fucosylated triantennaries (NA3FB, peak 9) were modestly elevated (P<0.05) and those of bigalacto core-α-1,6-fucosylated biantennary glycans (NA2F, peak 6) were decreased.

Callewaert et al and Liu et al previously published a structural analysis of these N-glycans [15,16]. The average relative abundance of these N-glycan structures is presented in Table 3. The abundance of the structures in peaks 1, 2, 3, 5, 6, 7, and 9 was significantly different in the GC, atrophic gastritis, and healthy control groups, indicating that different N-glycan patterns existed under various pathophysiological conditions. Compared with the healthy control group, peaks 1, 2, 5 and 9 were increased (P<0.05) and peaks 3, 6, and 7 were decreased in the GC group (P<0.001). The abundance of the structures in peaks 3, 5, 6, 7, and 9 was significantly different in the GC group compared with the atrophic gastritis group (Figure 2).

**Table 3:** N-Glycan profiling by DNA sequencer-assisted/fluorophore-assisted capillary electrophoresis.

	Means±SD				
Variable	Control (n=128)	Atrophic Gastritis (n=108)	GC (n=138)	*F*	*P*
Age, y^a^	50.10±5.95	52.11±6.29	51.01±6.01	0.27	0.766
Peak 1^a^	6.96±1.67	7.64±2.02	8.20±2.75	10.09	<0.001
Peak 2^a^	1.09±0.34	1.20±0.38	1.31±0.49	3.12	0.045
Peak 3^a^	6.28±1.63	6.47±1.38	5.76±1.28	10.48	<0.001
Peak 4^a^	5.76±0.93	5.61±0.81	5.75±0.82	1.12	0.327
Peak 5^a^	40.02±3.75	39.60±3.76	42.16±4.33	14.94	<0.001
Peak 6^a^	21.40±2.61	20.51±2.85	16.99±2.98	88.36	<0.001
Peak 7^a^	5.87±1.41	6.02±1.71	5.21±1.21	11.15	<0.001
Peak 8^a^	7.94±1.58	7.59±2.24	7.46±1.82	2.24	0.108
Peak 9^a^	2.33±0.92	2.59±1.55	4.00±1.77	48.12	<0.001
sumfuc^ab^	47.37±4.40	47.47±5.30	43.21±5.94	27.01	<0.001

Abbreviations: GC, gastric carcinoma; SD, standard deviation.

^a^ analyses of variance

^b^ Sumfuc represents the total abundance of α-1,6-fucosylated structures (the sum of peaks 1, 2, 3, 4, 6, and 7).

### 2.2: Construction and evaluation of a diagnostic model based on N-Glycan markers to differentiate gastric cancer patients from healthy controls

**Figure 3:**
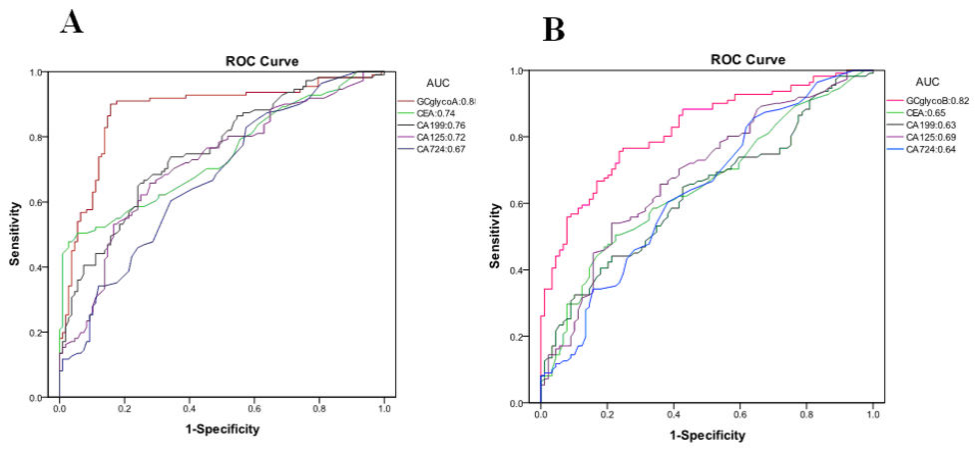
Receiver operating characteristic (ROC) curve analyses for the prediction of gastric carcinoma (GC). (A) The ROC analysis for distinguishing between GC and control subjects using an N-glycan marker-based GC diagnostic model (GCglycoA), CEA, CA19-9, CA125 or CA72-4. The areas under the ROC curve (AUCs) indicate the diagnostic power: CEA (0.74), CA19-9 (0.76), CA125 (0.72), CA72-4 (0.67) and GCglycoA (0.88). The diagnostic model was constructed by using forward stepwise logistic regression analysis: GCglycoA = -1.072+0.957peak4-0.331peak6+0.646peak9. (B) The ROC analysis for distinguishing between GC and atrophic gastritis using the GCglycoB diagnostic model, CEA, CA19-9, CA125 or CA72-4. The AUCs indicate the diagnostic power: GCglycoB (0.82), CEA(0.65), CA19-9 (0.63), CA125 (0.69) and CA72-4 (0.64). The diagnostic model was constructed by using forward stepwise logistic regression analysis: GCglycoB=5.273-1.371peak2+0.781peak4-0.453peak6+0.221peak9.

We evaluated the GC-related N-glycan alterations based on a logistic regression analysis. Logistic regression coefficients were utilized to estimate the odds ratios for each of the independent variables. The GCglycoA mathematic formula was constructed to differentiate the GC patients from the healthy controls (GCglycoA=-1.072+0.957*peak4-0.331*peak6+0.646*peak9). To assess the ability of GCglycoA, CEA, CA19-9, CA125 and CA72-4 to discriminate GC patients, we characterized the area under the ROC curves (AUC). Compared with CEA (AUC=0.74), CA19-9 (AUC=0.76), CA125 (AUC=0.72) and CA72-4 (AUC=0.67), GCglycoA more effectively distinguished GC patients from normal controls (AUC=0.88) in the training group (Figure 3A). Table 4 lists the sensitivity, specificity, PPV, NPV, and accuracy for the prediction of GC by CEA, CA19-9, CA125, CA72-4 and GCglycoA. CEA at the recommended value of 5.0 ng/mL had a sensitivity of 45.76%, 37 U/mL CA19-9 had a sensitivity of 38.14%, 40 U/mL CA125 had a sensitivity of 18.64%, and 9.8 U/mL CA72-4 had a sensitivity of 13.56%. An optimal cutoff value of -0.772 was selected for GCglycoA based on the ROC curve analysis. At this cutoff value, GCglycoA had a sensitivity of 75.42%, which is an increase in sensitivity of 29.66%, 37.28%, 56.78% and 61.86% compared with CEA, CA19-9, CA125 and CA72-4, respectively. The diagnostic accuracy of GCglycoA in differentiating GC patients from healthy controls increased by 10.62%, 16.82%, 25.67% and 28.76% compared with CEA, CA19-9, CA125 and CA72-4, respectively. When the model was applied to the retrospective validation group, the sensitivity increased 45.00%, 45.00%, 55.00% and 55.00% and the accuracy increased 15.00%, 17.50%, 20.00% and 20.00% compared with CEA, CA19-9, CA125 and CA72-4, respectively (Table 5).

**Table 4:** The Diagnostic Power for Differentiating Gastric Carcinoma from the Controls in the Training Group.

Cutoff value	Actual status, No. of subjects							
	Test	GC+	GC-	Sensitivity, %	Specificity, %	PPV, %	NPV, %	Accuracy, %
CEA (5 ng/mL)	GC+	54	5	45.76	95.37	91.53	61.68	69.47
	GC-	64	103					
CA19-9 (37 U/mL)	GC+	45	10	38.14	90.74	81.82	57.31	63.27
	GC-	73	98					
CA125 (40 U/mL)	GC+	22	7	18.64	93.52	75.86	51.27	54.42
	GC-	96	101					
CA72-4 (9.8 U/mL)	GC+	16	8	13.56	92.59	66.67	49.51	51.33
	GC-	102	100					
GCglycoA (-0.77)	GC+	89	16	75.42	85.19	84.76	76.03	80.09
	GC-	29	92					

Abbreviations: + positive; - negative; GC, gastric carcinoma; GCglycoA, N-glycan marker-based gastric cancer diagnostic model A; NPV, negative predictive value; PPV, positive predictive value.

**Table 5:** The Diagnostic Power for Differentiating Gastric Carcinoma from the Controls in the Retrospective Verification Group.

Cutoff value	Actual status, No. of subjects							
	Test	GC+	GC-	Sensitivity, %	Specificity, %	PPV, %	NPV, %	Accuracy, %
CEA (5 ng/mL)	GC+	8	0	40.00	100.00	100.00	62.50	70.00
	GC-	12	20					
CA19-9 (37 U/mL)	GC+	8	1	40.00	95.00	88.89	61.29	67.50
	GC-	12	19					
CA125 (40 U/mL)	GC+	6	0	30.00	100.00	100.00	58.82	65.00
	GC-	14	20					
CA72-4 (9.8 U/mL)	GC+	6	0	30.00	100.00	100.00	58.82	65.00
	GC-	14	20					
GCglycoA (-0.77)	GC+	17	3	85.00	85.00	85.00	85.00	85.00
	GC-	3	17					

Abbreviations: + positive; - negative; GC, gastric carcinoma; GCglycoA, N-glycan marker-based gastric cancer diagnostic model A; NPV, negative predictive value; PPV, positive predictive value.

The prospective evaluation indicated that the sensitivity improved 65.00%, 65.00%, 85.00% and 80.00% and the accuracy improved 30.00%, 27.50%, 37.50% and 37.50% compared with CEA, CA19-9, CA125 and CA72-4, respectively (Table 6). 

**Table 6:** The Diagnostic Power for Differentiating Gastric Carcinoma from the Controls in the Prospective Verification Group.

Cutoff value	Actual status, No. of subjects							
	Test	GC+	GC-	Sensitivity,%	Specificity,%	PPV, %	NPV, %	Accuracy,%
CEA (5 ng/mL)	GC+	6	1	30.00	95.00	85.71	57.58	62.50
	GC-	14	19					
CA19-9 (37 U/mL)	GC+	6	0	30.00	100.00	100.00	58.82	65.00
	GC-	14	20					
CA125 (40 U/mL)	GC+	2	0	10.00	100.00	100.00	52.63	55.00
	GC-	18	20					
CA72-4 (9.8 U/mL)	GC+	3	1	15.00	95.00	75.00	52.78	55.00
	GC-	17	19					
GCglycoA -0.77	GC+	19	2	95.00	90.00	90.48	94.74	92.50
	GC-	1	18					

Abbreviations: + positive; - negative; GC, gastric carcinoma; GCglycoA, N-glycan marker-based gastric cancer diagnostic model A; NPV, negative predictive value; PPV, positive predictive value.

### 2.3: Construction and evaluation of a diagnostic model to differentiate gastric cancer from atrophic gastritis

Using a logistic regression model, another diagnostic model (GCglycoB) was established to differentiate between GC and atrophic gastritis: GCglycoB=5.273-1.371*peak2+0.781*peak4-0.453*peak6+0.221*peak9. The optimal cutoff value for GCglycoB (0.594) was selected based on the ROC curve analysis. In the training group, the AUC for GCglycoB was 0.82, whereas the AUCs for CEA, CA19-9, CA125 and CA72-4 were 0.65, 0.63, 0.69 and 0.64, respectively (Figure 3B). The diagnostic accuracy of GCglycoB in differentiating GC from atrophic gastritis increased 21.26%, 24.64%, 31.40% and 34.30% and the sensitivity increased 27.97%, 35.59%, 55.09% and 60.17% compared with CEA, CA19-9, CA125 and CA72-4, respectively (Table 7). In the validation group, the 85.00% accuracy of GCglycoB represented an increase of 40.00%, 45.00%, 65.00% and 55.00% and the 85.00% sensitivity indicated an increase of 22.50%, 22.50%, 30.00% and 30.00% compared with CEA, CA19-9, CA125 and CA72-4, respectively (Table 8).

**Table 7:** The Diagnostic Power for Differentiating Gastric Carcinoma from Atrophic Gastritis in the Training Group.

Cutoff value	Actual status, No. of subjects							
	Test	GC+	GC-	Sensitivity, %	Specificity, %	PPV, %	NPV, %	Accuracy, %
CEA (5 ng/mL)	GC+	54	19	45.76	78.65	73.97	52.24	59.90
	GC-	64	70					
CA19-9 (37 U/mL)	GC+	45	17	38.14	80.90	72.58	49.66	56.52
	GC-	73	72					
CA125 (40 U/mL)	GC+	22	8	18.64	91.01	73.33	45.76	49.76
	GC-	96	81					
CA72-4 (9.8 U/mL)	GC+	16	8	13.56	91.01	66.67	44.26	46.86
	GC-	102	81					
GCglycoB (0.594)	GC+	88	8	73.73	91.01	91.58	72.32	81.16
	GC-	30	81					

Abbreviations: + positive; - negative; GC, gastric carcinoma; GCglycoB, N-glycan marker-based gastric cancer diagnostic model B; NPV, negative predictive value; PPV, positive predictive value.

**Table 8:** The Diagnostic Power for Differentiating Gastric Carcinoma from Atrophic Gastritis in the Retrospective Verification Group.

Cutoff value	Actual status, No. of subjects							
	Test	GC+	GC-	Sensitivity, %	Specificity, %	PPV, %	NPV, %	Accuracy, %
CEA (5 ng/mL)	GC+	9	4	45.00	80.00	69.23	59.26	62.50
	GC-	11	16					
CA19-9 (37 U/mL)	GC+	8	3	40.00	85.00	72.73	58.62	62.50
	GC-	12	17					
CA125 (40 U/mL)	GC+	4	2	20.00	90.00	66.67	52.94	55.00
	GC-	16	18					
CA72-4 (9.8 U/mL)	GC+	6	4	30.00	80.00	60.00	53.33	55.00
	GC-	14	16					
GCglycoB (0.594)	GC+	17	3	85.00	85.00	85.00	85.00	85.00
	GC-	3	17					

Abbreviations: + positive; - negative; GC, gastric carcinoma; GCglycoB, N-glycan marker-based gastric cancer diagnostic model B; NPV, negative predictive value; PPV, positive predictive value.

In the prospective group, GCglycoB was more accurate (85.00% vs. 30.00%, 30.00%, 10.00% and 15.00%) and sensitive (90.00% vs. 62.50%, 62.50%, 55.00% and 55.00%) than CEA, CA19-9, CA125 and CA72-4, respectively (Table 9). 

**Table 9:** The Diagnostic Power for Differentiating Gastric Carcinoma from Atrophic Gastritis in the Prospective Verification Group.

Cutoff value	Actual status, No. of subjects							
	Test	GC+	GC-	Sensitivity, %	Specificity, %	PPV, %	NPV, %	Accuracy, %
CEA (5 ng/mL)	GC+	6	1	30.00	95.00	85.71	57.58	62.5
	GC-	14	19					
CA19-9 (37 U/mL)	GC+	6	1	30.00	95.00	85.71	57.58	62.5
	GC-	14	19					
CA125 (40 U/mL)	GC+	2	0	10.00	100.00	100.00	52.63	55.00
	GC-	18	20					
CA72-4 (9.8 U/mL)	GC+	3	1	15.00	95.00	75.00	52.78	55.00
	GC-	17	19					
GCglycoB (0.594)	GC+	17	1	85.00	95.00	94.44	86.36	90.00
	GC-	3	19					

Abbreviations: + positive; - negative; GC, gastric carcinoma; GCglycoB, N-glycan marker-based gastric cancer diagnostic model B; NPV, negative predictive value; PPV, positive predictive value.

### 2.4: Decreased levels of total core-fucosylated proteins in gastric cancer

**Figure 4:**
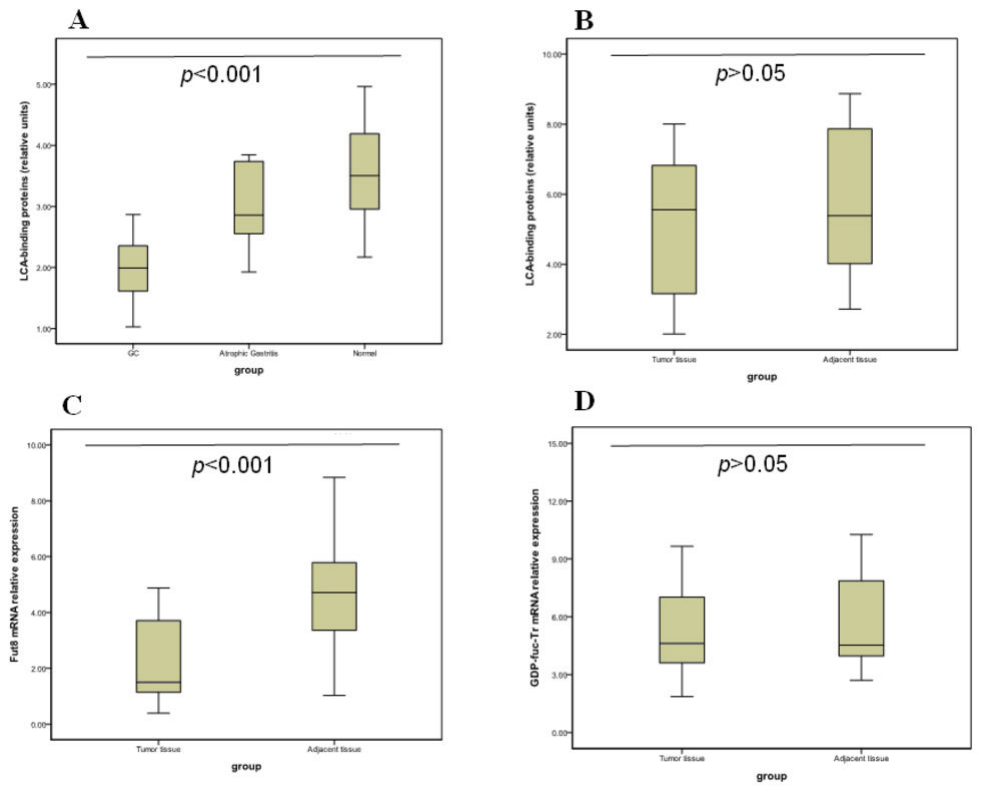
The abundance of total core fucosylated residues, α-1,6-fucosyltransferase (Fut8) and guanosine diphosphate (GDP)-fucose transporter (GDP-fuc-Tr) using lectin blotting and reverse transcription-polymerase chain reaction (RT-PCR). (A) Lectin blots of serum proteins probed with lens culinaris agglutinin A (LCA). The horizontal axis represents the experimental groups: control (n=20), atrophic gastritis (n=20), and gastric carcinoma (GC) (n=20); each pool consists of 3 homogenous samples. The vertical axis indicates the ratio of fucosylated protein to total protein. The difference between the groups was statistically significant (P <0.001). (B) Lectin blotting of tissue proteins probed with LCA. The horizontal axis represents the experimental groups: tumor tissue (n=20) and adjacent tissue (n=20). The vertical axis indicates the ratio of fucosylated protein to total protein. The difference between the groups was not statistically significant (P >0.05). (C) The relative messenger RNA (mRNA) expression of Fut8 in tissue as measured by RT-PCR. The horizontal axis represents the experimental groups: tumor tissue (n=20) and adjacent tissue (n=20). The vertical axis indicates the relative expression of Fut8. The difference between the groups was statistically significant (P <0.001). (D) The relative mRNA abundance of GDP-fuc-Tr in tissue as measured by RT-PCR. The horizontal axis represents the experimental groups: tumor tissue (n=20) and adjacent tissue (n =20). The vertical axis indicates the relative abundance of GDP-fuc-Tr. The difference between the groups was not statistically significant (P >0.05).

The total level of core α-1,6-fucose residues (the sum of peaks 1, 2, 3, 4, 6, and 7) was significantly lower (P<0.001; Table 2) in GC patients than in atrophic gastritis patients and normal controls. To confirm this finding, we evaluated the concentration of core fucosylated proteins in the serum and tissue of patients with GC using LCA because it specifically recognizes glycoproteins with α-1,6-fucosylated-N-acetyl-D-glucosamine-asparagine (GlcNAc-Asp) in the trimannosyl core. In serum, there were fewer LCA-binding core fucose residues in the GC group than in the atrophic gastritis and normal groups (Figure 4A). The total core fucose abundance was lower in GC tumors than in paired adjacent tissue (Figure 4B). To determine whether the change in total core fucosylation in GC tumors was related to altered glycosylation biosynthesis, the abundance of mammalian α-1,6-fucosyltransferase (Fut8) and guanosine diphosphate transporter (GDP-Tr) in GC tumors and adjacent tissue was analyzed by RT-PCR. The results revealed that Fut8 mRNA expression was lower in tumors than in adjacent tissue (Figure 4C). However, there was no significant difference in guanosine diphosphate (GDP)-fucose transporter (GDP-fuc-Tr) gene expression between tumors and adjacent tissue (Figure 4D).

### 2.5: Changes in N-Glycan markers before and after curative surgery

The abundance of the structures in peaks 1, 4, and 9 was significantly different after surgery compared with before surgery (Table 10). The results indicated that the increases in the core fucosylated peak (peak 3) and total core fucosylated N-glycans (sumfuc) were reversed after surgery. Ten weeks after surgery, the GCglycoA value became negative in 11 of the 40 patients with GC, as did the GCglycoB value in 22 of the 40 patients with GC.

**Table 10:** N-Glycan Profiling in Patients With Gastric Cancer Before and After Curative Surgery.

	Mean±SD or Median (interquartile range)		*T* -Test	
Variable	Before Surgery	After Surgery^a^	*T or Z*	*P*
Peak 1^b^	8.92±3.74	8.49±3.34	2.038	0.048
Peak 2^b^	1.24±0.59	1.52±1.74	-1.051	0.300
Peak 3^b^	5.96±1.52	6.14±1.33	-0.631	0.531
Peak 4^b^	5.73±0.69	5.21±1.01	2.602	0.013
Peak 5^b^	40.81±4.87	42.27±5.27	-1.513	0.138
Peak 6^b^	16.74±2.98	17.30±2.61	-0.809	0.423
Peak 7^b^	5.28±1.50	5.50±1.14	-0.716	0.478
Peak 8^b^	7.60±2.07	7.82±1.85	-0.586	0.562
Peak 9^b^	4.11±1.85	3.29±1.69	2.047	0.047
sumfuc^c^	43.88±7.77	44.16±6.10	-0.217	0.830
GCglycoA^d^	1.07 (0.49, 3.12)	0.28 (-0.90, 1.14)	-2.782	0.005
GCglycoB^d^	1.31 (0.56, 2.41)	0.41 (-0.54, 1.19)	-2.379	0.017

Abbreviations: GCglycoA and GCglycoB, N-glycan marker-based gastric cancer diagnostic models A and B, respectively; SD, standard deviation.

^a^ Ten weeks after surgery.

^b^ Paired-sample *T* test

^c^ Sumfuc indicates the total abundance ofα-1,6-fucosylated structures.

^d^ Wilcoxon signed-rank test.

### 2.6: Correlations between the diagnostic models and the clinical parameters

Currently, the tumor biomarkers CEA, CA19-9, CA125 and CA72-4 are widely used for the screening and monitoring of GC. Therefore, we determined the correlations between the individual N-glycan markers (including the diagnostic models), the tumor biomarkers and the TNM stage.

The results indicated that only the TNM stage was positively associated with GCglycoA (r=0.355, P <0.001) and GCglycoB (r=0.345, P <0.001) (Table 11). The abundance of the structures in peaks 6 and 9 was significantly different between the TNM stages (Table 12).

**Table 11:** The Correlation Between N-Glycan Markers and Pathobiochemical Parameters.

Correlation		CEA	CA19-9	CA125	CA72-4	TNM Stage	GCglycoA	GCglycoB
Peak 1								
	*r*	0.210^a^	0.127^a^	0.006^a^	-0.023^a^	0.088^a^	0.250^b^	0.055^b^
	*P*	0.016	0.148	0.944	0.793	0.316	0.004	0.532
Peak 2								
	*r*	0.009^a^	0.038^a^	0.011^a^	-0.067^a^	0.010^a^	0.224^b^	-0.099^b^
	*P*	0.917	0.667	0.904	0.445	0.906	0.010	0.258
Peak 3								
	*r*	0.083^a^	0.094^a^	0.066^a^	0.039^a^	-0.115^a^	-0.216^b^	-0.346^b^
	*P*	0.348	0.286	0.455	0.654	0.193	0.013	<0.001
Peak 4								
	*r*	^-^0.045^a^	0.160^a^	0.047^a^	-0.006^a^	-0.091^a^	0.122^b^	0.074^b^
	*P*	0.613	0.068	0.594	0.943	0.301	0.167	0.403
Peak 5								
	*r*	-0.029^a^	-0.156^a^	-0.020^a^	0.097^a^	0.108^a^	0.299^b^	0.478^b^
	*P*	0.743	0.076	0.816	0.270	0.218	0.001	<0.001
Peak 6								
	*r*	0.009^a^	0.020^a^	0.044^a^	-0.114^a^	-0.348^a^	-0.757^b^	-0.787^b^
	*P*	0.921	0.817	0.618	0.197	<0.001	<0.001	<0.001
Peak 7								
	*r*	-0.039^a^	0.089^a^	-0.075^a^	-0.161^a^	-0.245^a^	-0.368^b^	-0.517^b^
	*P*	0.658	0.311	0.394	0.066	0.005	<0.001	<0.001
Peak 8								
	*r*	-0.135^a^	-0.058^a^	0.002^a^	-0.087^a^	-0.035^a^	-0.215^b^	0.011^b^
	*P*	0.124	0.513	0.984	0.321	0.692	0.014	0.897
Peak 9								
	*r*	0.028^a^	-0.072^a^	-0.034^a^	0.095^a^	0.315^a^	0.794^b^	0.591^b^
	*P*	0.751	0.413	0.703	0.280	<0.001	<0.001	<0.001
GCglycoA								
	*r*	-0.033^b^	-0.042^b^	-0.012^b^	0.046^b^	0.355^b^	1	0.869^b^
	*P*	0.710	0.637	0.896	0.605	<0.001		<0.001
GCglycoB								
	*r*	-0.052^b^	-0.045^b^	0.027^b^	0.105^b^	0.345^b^	0.869^b^	1
	*P*	0.558	0.611	0.760	0.232	<0.001	<0.001	
Sumfuc^c^								
	*r*	0.061^a^	0.151^a^	0.040^a^	-0.108^a^	-0.170^a^	-0.353^b^	-0.580^b^
	*P*	0.486	0.085	0.651	0.221	0.052	<0.001	<0.001

Abbreviations: r, correlation coefficient.

^a^ Pearson’s correlation analysis

^b^ Spearman’s correlation analysis

^c^ Sumfuc indicates the total abundance ofα-1,6-fucosylated structures.

**Table 12:** N-Glycan Profiling in Patients With Gastric Carcinoma Based on TNM Stage.

	Mean ± SD					
Variable	Stage I (n=22)	Stage II (n=28)	Stage III (n=63)	Stage IV (n=25)	*F*	*P*
Peak 1^a^	7.94±1.87	7.83±3.21	8.21±2.54	8.82±3.44	0.600	0.616
Peak 2^a^	1.21±0.38	1.24±0.51	1.23±0.42	1.67±0.49	1.108	0.348
Peak 3^a^	6.09±1.16	5.75±1.25	5.61±1.12	5.83±1.79	0.770	0.513
Peak 4^a^	5.81±0.85	5.97±0.74	5.63±0.79	5.78±0.96	1.116	0.345
Peak 5^a^	40.36±4.10	42.25±4.45	43.12±4.21	41.31±4.28	2.646	0.052
Peak 6^a^	19.66±3.09	17.13±2.45	16.33±2.68	15.99±2.78	9.258	<0.001
Peak 7^a^	5.72±1.17	5.30±1.05	5.14±1.25	4.82±1.22	2.312	0.079
Peak 8^a^	7.49±1.74	7.43±1.88	7.41±1.73	7.58±2.18	0.049	0.986
Peak 9^a^	2.67±1.58	3.97±1.80	4.17±1.33	4.84±2.27	6.834	<0.001
Sumfuc^b^	46.43±4.77	43.21±5.55	42.14±6.94	42.91±6.94	2.944	0.036

Abbreviations: SD, standard deviation.

^a^ analyses of variance

^b^ Sumfuc indicates the total abundance of α-1,6-fucosylated structures.

## Discussion

Glycosylation is one of the most common post-translational modifications. Changes in N-linked glycosylation occur during the development of various diseases [17]. Among the various methods for identifying novel serum glycobiomarkers [18,19], DSA-FACE is a simple and efficient technology for measuring changes in N-glycans in biological fluids, such as serum. We previously used this technology to support the diagnosis of HCC and colorectal cancer [13]. In the current study, we used DSA-FACE to analyze N-linked glycans and to identify the specific N-glycan profile during the development of gastric cancer.

Clinically, there are no reliable tumor biomarkers for gastric cancer, but CEA, CA19-9, CA125 and CA72-4 are commonly used to screen for this disease. However, they do not provide sufficient sensitivity or reliability for early detection. CEA is a highly heterogeneous glycoprotein that contains 60% carbohydrate. Individual differences in glycoprotein patterns are expected. Therefore, we hypothesized that N-glycan profiling would be more effective than the detection of individual glycosylated molecules. 

Based on the statistical analyses and forward stepwise logistical regression after performing serum DSA-FACE, we constructed 2 diagnostic models: GCglycoA (for distinguishing between GC and control) and GCglycoB (for distinguishing between GC and atrophic gastritis). In the training group, both models had a better diagnostic performance (accuracy >80.0%) than CEA, CA19-9, CA125 or CA72-4. We confirmed that the diagnostic values of GCglycoA and GCglycoB were greater than those of CEA, CA19-9, CA125 and CA72-4 in a retrospective validation group and in an independent prospective cohort. 

In analyzing the abundance of individual N-glycan structures, we determined that the abundance of core-fucosylated structures decreased significantly in gastric cancer. This is similar to what occurs in colorectal cancer [20] but differs from what has been reported for other cancers, such as the increased core fucosylation of alpha-fetoprotein that was observed in HCC [21,22]. To validate our findings, we performed lectin blotting of tissue and serum samples. Using HCC serum as a positive control, we observed decreased N-glycan fucosylation in the lectin blots of GC serum samples. A similar trend was present in lectin blots of GC tissue.

Protein fucosylation is regulated by several fucosyltransferases, the GDP-fucose synthetic pathway, and GDP-fuc-Tr [23]. GDP-fuc-Tr is important for GDP-fucose transport. Core fucosylation is catalyzed by Fut8. We determined the expression of Fut8 and GDP-fuc-Tr to analyze the relevant steps for altering core fucosylation. The results indicated that Fut8 gene expression was significantly lower in tumors than in adjacent tissue and that GDP-fuc-Tr gene expression did not differ significantly between tumor and adjacent tissue. The results indicated that decreased Fut8 expression could be responsible for the reduced core 1,6-fucosylation in GC.

Previously, we determined that peak 9 (a branching α-1,3-fucosylated triantennary glycan, NA3Fb) was associated with the development of HCC and colorectal cancer [9,21]. Notably, peak 9 was also significantly increased in patients with gastric cancer. N-acetylglucosaminyltransferase V (GnT-V) is responsible for the increase in multiantennary glycans (NA3Fb, peak 9) [24]. This suggested that GnT-V might be involved in malignant transformation, and the molecular mechanism by which N-glycosylation patterns are altered in gastric cancer remains a critical challenge for future studies.

## Conclusions

We concluded that serum N-glycan profile was altered during the development of gastric cancer. The serum N-glycan might aid clinicians to diagnose gastric cancer in its earliest stages and would also contribute to distinguish it from other similar disorders. Our study provided additional insight into the molecular and cellular biology of gastric cancer by confirming that glycosylation is involved in the pathogenesis of cancer. A future glycobiological approach will focus on the changes in glycosylation that accompany each step of cancer development and progression to improve the diagnosis, monitoring, and screening of gastric cancer.
